# Clonal Immune Responses of Mycobacterium-Specific γδ T Cells in Tuberculous and Non-Tuberculous Tissues during *M. tuberculosis* Infection

**DOI:** 10.1371/journal.pone.0030631

**Published:** 2012-02-01

**Authors:** Dan Huang, Crystal Y. Chen, Meihong Zhang, Liyou Qiu, Yun Shen, George Du, Keyuan Zhou, Richard Wang, Zheng W. Chen

**Affiliations:** 1 Department of Microbiology and Immunology, Center for Primate Biomedical Research, University of Illinois College of Medicine, Chicago, Illinois, United States of America; 2 Center for Gene Diagnosis, Zhongnan Hospital, Wuhan University, Wuhan, China; 3 Key Laboratory of Medical Molecular Activity Research, Guangdong Medical College, Dongguan, China; 4 College of Life Science, Henan Agricultural University, Zhengzhou, China; University of Maryland, United States of America

## Abstract

**Background:**

We previously demonstrated that unvaccinated macaques infected with large-dose *M.tuberculosis*(Mtb) exhibited delays for pulmonary trafficking of Ag-specific αβ and γδ T effector cells, and developed severe lung tuberculosis(TB) and “secondary” Mtb infection in remote organs such as liver and kidney. Despite delays in lungs, local immunity in remote organs may accumulate since progressive immune activation after pulmonary Mtb infection may allow IFNγ-producing γδ T cells to adequately develop and traffic to lately-infected remote organs. As initial efforts to test this hypothesis, we comparatively examined TCR repertoire/clonality, tissue trafficking and effector function of Vγ2Vδ2 T cells in lung with severe TB and in liver/kidney without apparent TB.

**Methodology/Principal Findings:**

We utilized conventional infection-immunity approaches in macaque TB model, and employed our decades-long expertise for TCR repertoire analyses. TCR repertoires in Vγ2Vδ2 T-cell subpopulation were broad during primary Mtb infection as most TCR clones found in lymphoid system, lung, kidney and liver were distinct. Polyclonally-expanded Vγ2Vδ2 T-cell clones from lymphoid tissues appeared to distribute and localize in lung TB granuloms at the endpoint after Mtb infection by aerosol. Interestingly, some TCR clones appeared to be more predominant than others in lymphocytes from liver or kidney without apparent TB lesions. TCR CDR3 spetratyping revealed such clonal dominance, and the clonal dominance of expanded Vγ2Vδ2 T cells in kidney/liver tissues was associated with undetectable or low-level TB burdens. Furthermore, Vγ2Vδ2 T cells from tissue compartments could mount effector function for producing anti-mycobacterium cytokine.

**Conclusion:**

We were the first to demonstrate clonal immune responses of mycobacterium-specific Vγ2Vδ2 T cells in the lymphoid system, heavily-infected lungs and lately subtly-infected kidneys or livers during primary Mtb infection. While clonally-expanded Vγ2Vδ2 T cells accumulated in lately-infected kidneys/livers without apparent TB lesions, TB burdens or lesions appeared to impact TCR repertoires and tissue trafficking patterns of activated Vγ2Vδ2 T cells.

## Introduction

Tuberculosis(TB) remains one of the major causes of global morbidity and mortality, and has become increasingly prevalent and deadly as a result of HIV/AIDS pandemic and the emergence of extensively drug resistant (XDR) strains of *M. tuberculosis* [1], [2]. Elucidation of essential components of immunity against TB in humans may help to design better vaccines or immunotherapeutics for ultimate control of global TB pandemics [3]. Whereas studies in murine TB model show that Th1 cells and IFNγ or TNFα are important for protection against active Mtb infection [4], [5], the role of CD4+ T cells in anti-TB immunity is also implicated in HIV-infected humans and SIVmac-infected macaques [6], [7]. Moreover, the contribution of memory CD8+ T cells to resistance to TB has recently been demonstrated in nonhuman primates [8]. Nevertheless, a correlation between blood Th1 cells or IFNγ and anti-TB immunity in HIV-negative individuals has not been found [4]. Recent mechanistic studies of BCG vaccine-induced anti-TB immunity in macaques suggest that rapid pulmonary trafficking and accumulation of vaccine-elicited CD4+ and CD8+ T effector cells after Mtb infection may be one of immune mechanisms for protection [9]. However, while >90% of humans resist to active TB after Mtb exposure [10], little is known about how human immune cells control a primary Mtb infection. Investigating primary immune responses of various helper T cells and cytotoxic T cells including antigen-specific γδ T cells may help to identify effective or orchestrated immunity to primary Mtb infection.

Mycobacterium-specific Vγ2Vδ2 T cells exist only in humans and nonhuman primates. Accumulating evidence suggests that Vγ2Vδ2 T cells can contribute to both innate and adaptive immune responses in infections [11]. We and others have demonstrated that Vγ2Vδ2 T cells can be specifically activated and expanded by phosphoantigen (*E*)-4-hydroxy-3-methyl-but-2-enyl pyrophosphate (HMBPP) produced by Mtb and other selected pathogens, and that soluble Vγ2Vδ2 TCR can bind to HMBPP presented by APC [11], [12], [13], [14], [15]. Importantly, Vγ2Vδ2 T cells can mount major expansion during mycobacterium infections, and rapid recall-like expansion of these γδ T cells after Mtb challenge of BCG-vaccinated macaques is associated with BCG-induced protection against fatal TB in juvenile rhesus macaques [11]. Furthermore, major expansion of Vγ2Vδ2 T effector cells after HMBPP+ IL-2 post-challenge treatment can also lead to homeostatic protection against severe pneumonic plague lesions after inhalational *Y. pestis* infection of macaques [16]. However, a definitive role of Vγ2Vδ2 T cells in anti-TB immunity remains to be determined, and the definition requires in-depth studies of immune biology of these HMBPP-specific Vγ2Vδ2 T cells in primary Mtb infection.

Clonal immune responses of Vγ2Vδ2 T cells and their contribution to resistance to TB during primary Mtb infection remain unknown. Addressing this question may require an optimal model system for Mtb infection. We previously demonstrated that in contrast to vaccine-protected nonhuman primates, unvaccinated juvenile macaques infected with large-dose Mtb by aerosol exhibited delays for development and pulmonary trafficking of Ag-specific αβ and γδ T effector cells producing IFNγ, and developed profound inflammatory responses and severe TB lesions in lungs [8], [9], [11], [17]. Notably, severe lung TB resulted in transient extrathoracic Mtb dissemination, and a late or “secondary” Mtb infection in remote organ kidney or liver without apparent TB lesions [18]. Progressive immune activation after initial pulmonary Mtb infection may allow IFNγ-producing γδ and αβ T cells to timely develop in response to a late extrathoracic Mtb infection, and selectively traffic to the subsequently-infected remote organ liver or kidney for mounting potential local immunity. As initial efforts to test this presumption, we conducted comparative studies of TCR repertoire/clonality, tissue trafficking and effector function of Vγ2Vδ2 T cells in unprotected lung and in “lesions-free” remote organ kidney or liver. We focus on Vγ2Vδ2 T cells because these cells can readily undergo trans-endothelial mucosal migration after activation and expansion in lymphoid system [15].

## Results

### Broad TCR repertoire for Vγ2Vδ2 T-cell subpopulation in lymphoid system during primary Mtb infection of macaques

We previously demonstrated that mycobacterium-specific Vγ2Vδ2 T cells could expand in lymphoid tissues, and traffic to and accumulate in the interstitial compartment of non-lymphoid tissues at 1–1.5 months after Mtb infection by aerosol route [18]. However, little is known about TCR repertoire of these antigen-specific γδ T cells and their tissue trafficking patterns during infection. As an initial comparative study, we examined the clonality and TCR repertoires of Vγ2Vδ2 T cells in the blood and spleens during Mtb infection of macaques. We chose spleens as representative lymphoid tissues in this study as spleen tissues accommodated larger increases in Vγ2Vδ2 T cells than lymph nodes in TB [18] and were more readily available at necropsy.

**Figure 1:**
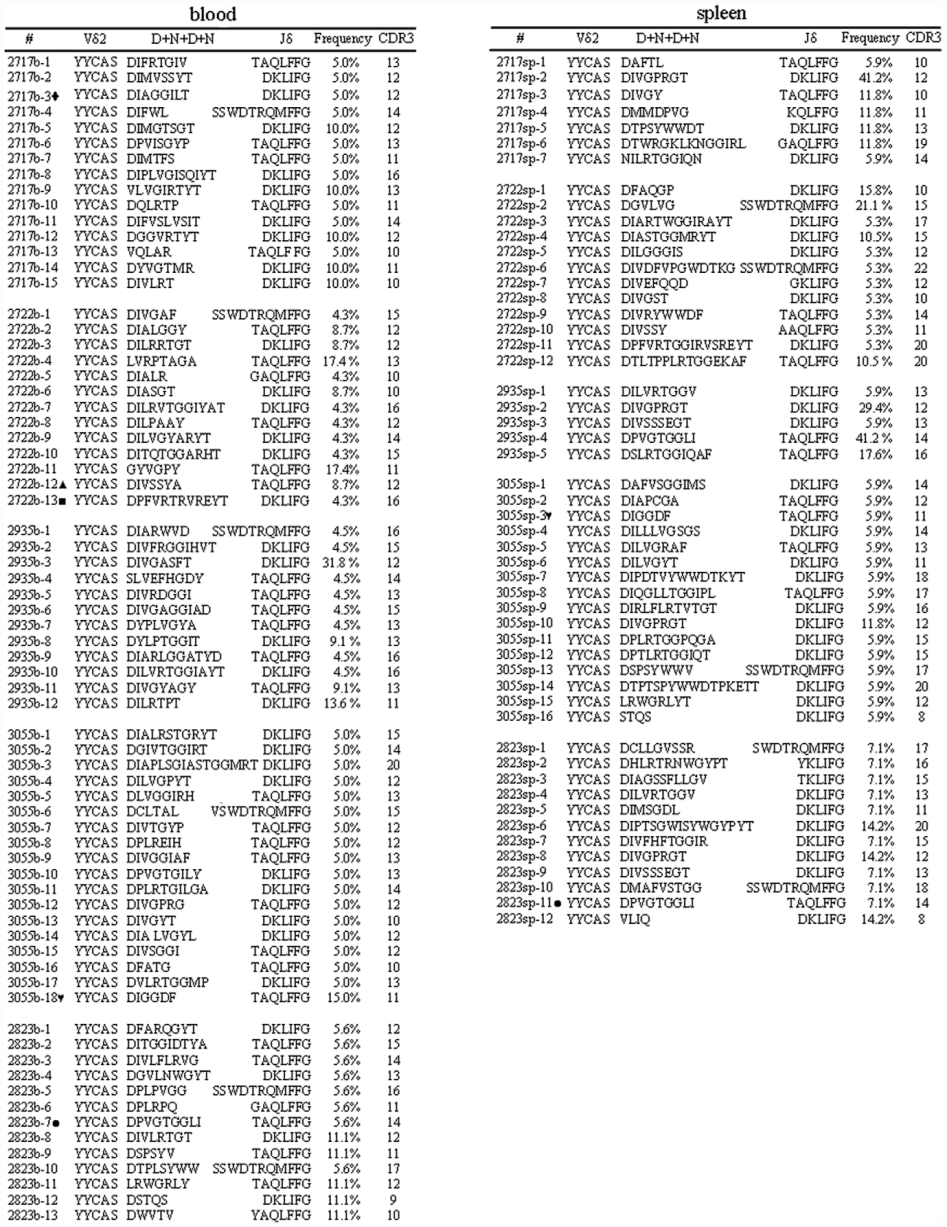
Broad T cell repertoire in Vγ2Vδ2 T-cell subpopulation in lymphoid system during primary Mtb infection of macaques. Shown are individual Vδ2 TCR clones isolated from PBL (left) and lymphocytes of spleen tissues (right) from 5 Mtb-infected macaques. The flow cytometry data indicating cellular expansion of Vγ2Vδ2 T cells in spleen were described in the text. Note that spleen lymphocytes in which major expansion of Vγ2Vδ2 T cells was seen were used for RNA isolation, cDNA synthesis and Vδ2 TCR sequence analyses. Note polyclonal sequences of Vδ2 TCR in cDNA derived from spleen lymphocytes and PBLs. Frequencies were expressed as the number of individual clones among the total analyzed clones. Similar data indicating polyclonal representation of Vγ2Vδ2 T cells in PBL before Mtb infection were also seen (data not shown). CDR3 were presumably indicated based on the definition for TCR β CDR3 [9]. D indicates diversity region; N indicates non-determining region of TCR receptor genes. Clones marked by ‘♦’ were present in the blood, lung and kidney (2717). Clones marked by ‘
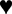
’ and ‘•’ were present in the blood, lung and spleen(3055 and 2823). Clones marked by ‘▴’ and ‘▪’ were present in the blood and lung(2722).

There was no apparent expansion of blood Vγ2Vδ2 T cells overtime after Mtb infection [18]. This appeared to be relevant to the infection route and Mtb bacterial burden, as intravenous infection of macaques with mycobacteria led to high bacterial burden in the blood or systemic site and induced major expansion of blood Vγ2Vδ2 T cells [7], [18], [19]. Blood Vγ2Vδ2 T cells displayed polyclonal Vδ2-bearing TCR sequences at 1–1.5 month after pulmonary Mtb infection (Fig. 1). The broad Vδ2 TCR repertoire was also seen in the blood circulation before infection([11] and data not shown). Interestingly, whereas Vγ2Vδ2 T cells in spleens expanded to the level of 15±5% (means± SD) in total CD3+ T cells at 1–1.5 month after pulmonary Mtb infection (<2% in naïve controls, [18]), these expanded γδ T cells expressed remarkable polyclonal Vδ2 TCR sequences (Fig. 1). The Vδ2 TCR repertoire appeared to be quite broad because most clones isolated from spleen tissues of Mtb-infected macaques were not seen in the clonotypic TCR sequences identified in the blood circulation at the time these macaques developed severe TB (Fig. 1). Three different Jδ segments were employed by the TCR clones (Fig. 1). However, some TCR clones were more frequently present in expanded Vγ2Vδ2 T cells isolated from spleen tissues of the infected macaques. For examples, clone #2 from macaque 2717 or clones #2 and #4 from macaque 2935 accounted for almost ∼50% of the total Vδ2 TCR clones identified in splenic Vγ2Vδ2 T cells, and a number of subdominant clones (frequencies were 10–15%) were also seen in splenic Vδ2 TCR clones from three infected macaques (Fig. 1). Thus, these results suggested that Vγ2Vδ2 TCR repertoire in lymphoid system remained broad after pulmonary Mtb infection, with polyclonal expansion of HMBPP-specific Vγ2Vδ2 T cells in spleen tissues.

### Polyclonally-expanded Vγ2Vδ2 T cells from lymphoid tissues appeared to distribute and localize in lung TB granuloms after Mtb infection by aerosol

**Figure 2:**
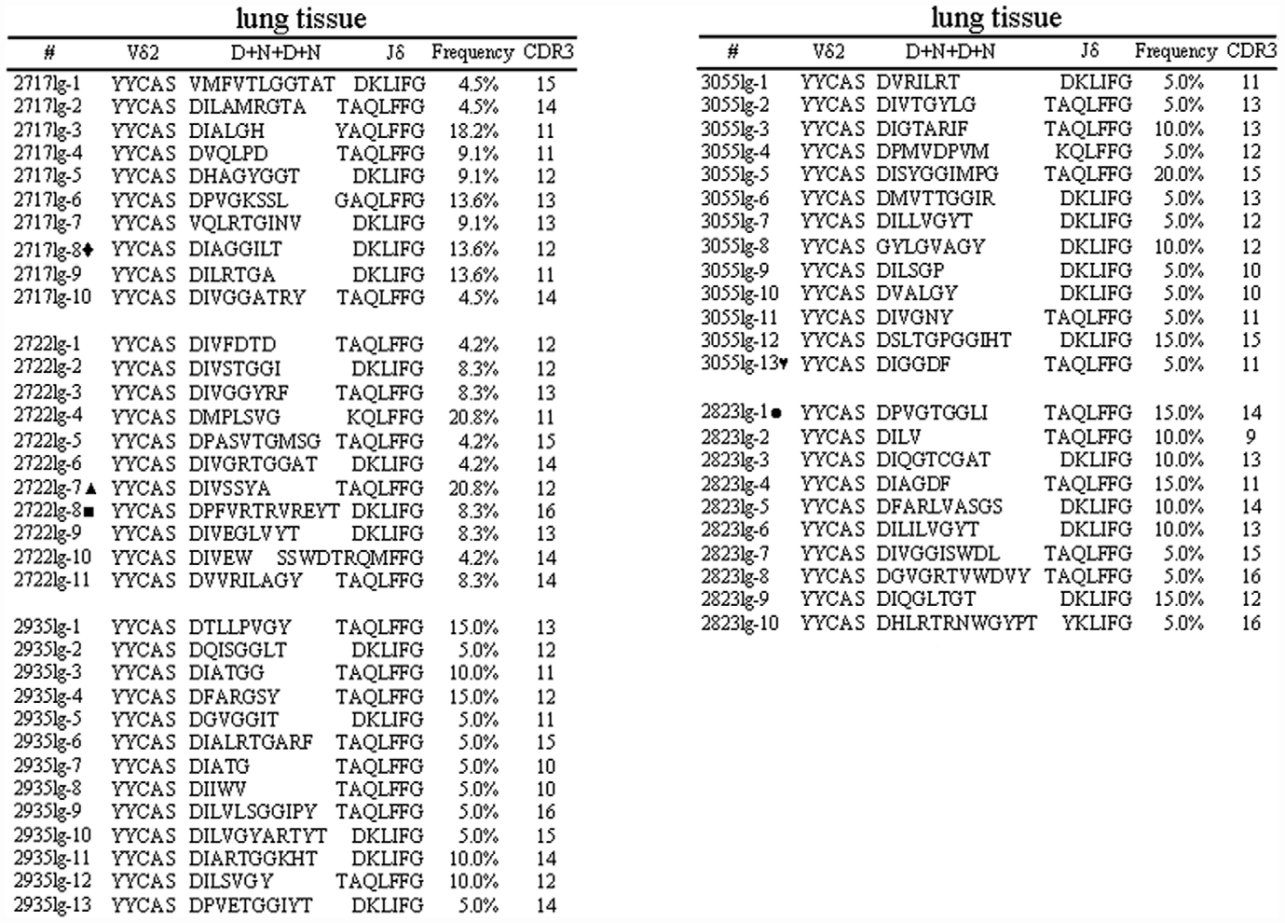
Polyclonally-expanded Vγ2Vδ2 T cells from lymphoid tissues appeared to distribute and localize in lung TB granuloms after Mtb infection by aerosol. Shown are individual Vδ2 TCR clones isolated from lymphocytes of lung tissues from five Mtb-infected macaques. The immunohistochenistry data showing infiltration and distribution of Vγ2Vδ2 T cells in TB granulomas were shown in the previous publication [18]. Flow cytometry data indicating cellular expansion of Vγ2Vδ2 T cells in CD3+ T cells isolated from the lung tissues were described in the text. Note polyclonal Vδ2 TCR sequences and sub-dominant clones in cDNA derived from lung lymphocytes in which expansion of Vγ2Vδ2 T cells was detected. Clones present in blood and spleen were marked as described in the legend of Fig. 1.

We then sought to examine TCR repertoire and potential clonotypic trafficking for expanded Vγ2Vδ2 T cells in the infection site, lung tissue, after Mtb infection by aerosol. Apparently, many Vγ2Vδ2 T cells distributed and accumulated in TB granulomas lesions after Mtb infection by aerosol [18], and Vγ2Vδ2 T cells comprised 23±5% of total CD3+ T cells isolated from lung tissues ([18], <2% of lung T cells in naïve controls). Notably, Vδ2 TCR clones identified in pulmonary Vγ2Vδ2 T cells were polyclonal, with many distinct clonotypes (Fig. 2). Despite such diverse TCR repertoire, however, a number of clones that sub-dominantly expanded in lymphoid tissues appeared to distribute in lungs since they were repeatedly detected with frequencies of 15–20% in all the clones isolated from lung tissues (Fig. 2). Moreover, five of these pulmonary clones were also present in the blood circulation (macaques 2717, 2722, 3055, 2823) or in both blood and spleen(3055,2823), or in both blood and kidney(2717), implicating that these subdominant clones had trafficked to lung tissues from the lymphoid system after Mtb infection by aerosol. These results therefore suggested that polyclonally-expanded Vγ2Vδ2 T cells from lymphoid tissues appeared to distribute and localize in severe lung TB tissues after Mtb infection by aerosol route.

### Some Vδ2 T cell clones of Vγ2Vδ2 T-cell subpopulation appeared to be more predominant than others in lately Mtb-infected liver or kidney tissue

The next immunological question was whether patterns of Vγ2Vδ2 T cell repertoire in remote organs(away from the lung infection site) were different from lymphoid system or early- and heavily-infected lungs. We chose kidney and liver as representative remote non-lymphoid organs as a late and subtle Mtb infection was anticipated in these organs after transient extrathoracic Mtb dissemination due to severe lung TB [18], and as progressively-activated Vγ2Vδ2 T effector cells after initial pulmonary exposure to Mtb might exhibit different TCR repertoires or trafficking patterns in timely response to a late Mtb infection of kidney or liver.

**Figure 3:**
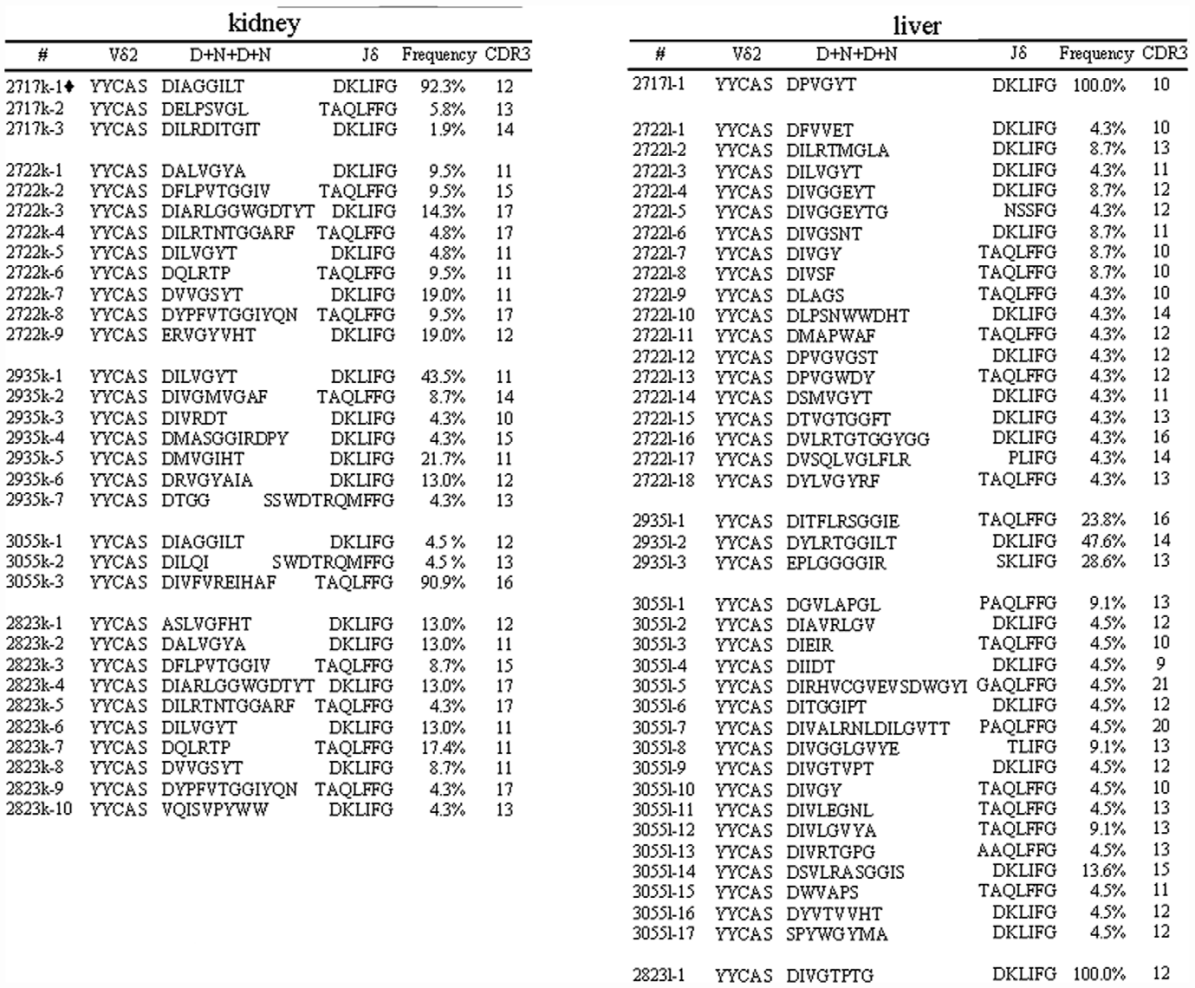
Some Vδ2 T cell clones of Vγ2Vδ2 T-cell subpopulation appeared to be more predominant than others in lately Mtb-infected liver or kidney. The localization of Vγ2Vδ2 T cells in interstitial tissues of kidney or liver were shown in the previous publication [18]. Expansion of Vγ2Vδ2 T cells in CD3+ T cells isolated from the kidney or liver tissues were described in the text. Note that three macaques(2717, 3055, 2935) exhibited dominance of a single clone or oligo-clones bearing a same length of CDR3 in TCR cDNA derived from kidney lymphocytes in which expansion of Vγ2Vδ2 T cells was detected. In cDNA derived from liver lymphocytes, a dominance of a single TCR clone or clones with a restricted CDR3 length was also noted in three macaques (2717, 2823, 2935). Clones marked by ‘♦’were present in the blood,lung and kidney(2717).

We found that Vγ2Vδ2 T cells trafficked to and localized in endothelia-interstitial tissue interface of liver and kidney in response to lately Mtb infection [18]. The late Mtb infection led to increases in numbers of Vγ2Vδ2 T cells to 25% and 32% of total CD3+ T cells in liver and kidney, respectively, whereas in naïve control macaques Vγ2Vδ2 T cells comprised <2% of total CD3+ T cells isolated from kidney or liver tissues [18]. Surprisingly, expanded Vγ2Vδ2 T cells in these remote organs from three of five macaques exhibited dominance of a single clone or oligo-clones bearing a selected CDR3 length, although one macaque (2722) did not show such predominance (Fig. 3). In fact, a single dominant clone could comprise >90% of 52 cDNA clones derived from Vγ2Vδ2 T cells in kidney tissues of macaques 2717 and 3055(Fig. 3). Macaque 2935 exhibited two dominant clones bearing 11 aa in CDR3, which accounted for ∼65% of all clones identified in the kidney tissues (Fig. 3). While macaque 2823 exhibited polyclonal TCR clonotypes, 4 sub-dominant clones bearing 11 aa in CDR3 comprised ∼52% of all the clones identified in the kidney (Fig. 3).

Similarly, expanded Vγ2Vδ2 T cells in liver tissues from four infected macaques also displayed dominance of a single TCR clone or clones with a restricted CDR3 length (Fig. 3). Virtually, only one single clonotypic TCR sequence was found in cDNA clones isolated from the expanded Vγ2Vδ2 T cells in liver tissues of the macaques 2717 and 2823; one single clonotypic TCR was found accounting for almost 50% of all the clones derived from the liver of the macaque 2935 (Fig. 3). Although expanded Vγ2Vδ2 T cells from the macaque 3055 showed polyclonal Vδ2 TCR sequences, ∼36% of these clones shared a same CDR3 length, 13 aa (Fig. 3).

Furthermore, we sought to determine whether Vγ2Vδ2 T-cell subpopulation in remote tissues were more clonally dominated or restricted than in blood/spleen or lung. We employed two-tailed Fisher exact test, as previously described [20], [21], to examine whether there were significant differences in dominant Vδ2 clonotypes (perturbation of TCR repertoire) between blood, spleen, lung, liver and kidney tissue compartments. We found that Vδ2 TCR repertoires in liver and kidney tissues were more clonally dominated or significantly perturbed when compared to those in the blood/lung and spleen (Table 1). The TCR repertoire in spleen was more clonally dominated than that in the blood and lung (Table 1).

**Table 1:** P values derived from statistical analyses of frequencies of dominant Vδ2 clonotypes between different tissues compartments (n = 5).

	Spleen	Lung	Liver	Kidney
Blood, vs#	0.0041, **	0.4855	0.0001, ***	0.0001, ***
Spleen, vs		0.0238, *	0.0001, ***	0.0001, ***
Lung, vs			0.0001, ***	0.0001, ***
Liver, vs				0.3726

# p value = 0.0041(**, very significant) when frequencies of dominant Vδ2 clonotypes in blood were compared with those in spleen(Blood vs Spleen). Individual dominant Vδ2 clonotypes were defined if they comprised >20% of the clones identified in a tissue compartment or blood from a macaque. Frequencies of dominant Vδ2 clonotypes among total TCR clones in a compartment from five macaques (Figs. 1, 2, 3) were calculated and analyzed for statistical significance between different tissue compartments using two-tailed Fisher exact test. We also statistically compared percentage numbers for total distinct Vδ2-bearing clones between different tissues compartments (n = 5), and found similar trends of results suggesting that Vδ2 repertoires in blood and lung were significantly broader than those in liver and kidney(data not shown).

Taken together, some Vδ2 T cell clones of Vγ2Vδ2 T-cell subpopulation appeared to be more predominant than others in lately Mtb-infected liver or kidney.

### TCR CDR3 spetratyping revealed predominance of a selected CDR3 length in expanded Vγ2Vδ2 T cells in kidney/liver tissue compartments in “secondary” local Mtb infection

**Figure 4:**
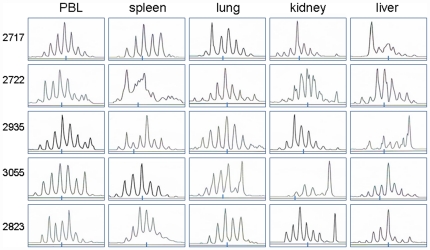
TCR CDR3 spetratyping revealed predominance of a selected CDR3 length in expanded Vγ2Vδ2 T cells in kidney/liver tissue compartments in late local Mtb infection. Shown are the Vδ2 TCR CDR3 profiles revealed by Genescan-based spectratyping as previously described [22]. The numbers of nucleotides in the different CDR3 lengths were determined in control experiments [22], and were expressed as predicted numbers of amino acids. A short line at the bottom of each histogram represents the predicted CDR3 length of 12 aa. The profiles of CDR3 lengths in Vδ2 TCR cDNA from blood, spleen and lung of the Mtb-infected macaques were diverse without a restricted selection of a single CDR3 length. In contrast, Vδ2 TCR cDNA derived from expanded Vγ2Vδ2 T cells in kidney or liver tissues of four infected macaques exhibited predominance of a selected CDR3 length. A selected CDR3 length was consistent with the clonal dominance of TCR sequence analyses in Fig. 3.

The dominance of a single TCR clone or CDR3 length at sequences levels prompted us to perform TCR CDR3 spetratyping, as we previously did [22], for an additional TCR repertoire analysis. Notably, the profiles of CDR3 lengths in Vδ2 TCR cDNA from blood, spleen and lung of the Mtb-infected macaques were diverse without a restricted selection of a single CDR3 length (Fig. 4). Overall, the occurrence of multiple CDR3 lengths in these tissues was consistent with polyclonal representations of clonotypic TCR sequences as seen in conventional cloning/sequencing analyses (Figs. 1,2,3). In contrast, Vδ2 TCR cDNA derived from expanded Vγ2Vδ2 T cells in kidney and liver tissues of four infected macaques could exhibit predominance of a selected CDR3 length (Fig. 4). These selected individual CDR3 lengths appeared to correspond to the clonotypic CDR3 sequences(VDDJ) as shown in Fig. 3. In fact, the predominant CDR3 lengths predictive of 13 aa, 11 aa and 16 aa detected in Vδ2 TCR cDNA from kidney tissues of three macaques 2717, 2935 and 3055 were explainable by the dominant TCR clones bearing the same CDR3 sequence lengths found in these animals, respectively (Fig. 3). Similarly, the predominant CDR3 lengths of 10 aa, 16aa and 12aa from liver tissues of macaques 2717, 2935 and 2823 were consistent with those dominant TCR clones bearing the same CDR3 lengths in these animals, respectively (Fig. 3). For the macaque 3055, a predominant CDR3 length predictive of 13 aa was revealed in Vδ2 TCR cDNA from the liver, which was not contradictory to the found polyclonal Vδ2 TCR sequences as 5 different TCR clones accounting for almost ∼40% of the total clones shared a same CDR3 length of 13 aa(Fig. 3). Subtle, focal Mtb infection might contribute to the predominance of a single TCR clone or CDR3 length in the kidney or liver, because unexpanded Vγ2Vδ2 T cells from kidney or liver tissues of naïve control macaques did not exhibit dominance of a selected CDR3 length(data not shown). Thus, the results from CDR3 spetratyping analyses supported the frequency data of Vδ2 TCR sequences, suggesting that Vγ2Vδ2 T cells accumulating in kidney and liver tissues after late or secondary infection could exhibit either dominance of single TCR clone/CDR3 length or polyclonal representation

### Clonal dominance of expanded Vγ2Vδ2 T cells in lately-infected kidney/liver tissues was associated with undetectable or low-level TB burdens

The trend for clonal dominance of expanded Vγ2Vδ2 T cells in kidneys and livers but not in lungs after pulmonary Mtb infection raised a question as to whether TB burden could impact TCR repertoire and trafficking patterns of Vγ2Vδ2 T cells in non-lymphoid tissues. The severe TB lesions (extensive caseating and miliary lesions or caseation pneumonia) and high-level TB burdens (>7000 bacilli organisms per 10 mg tissue cells) in lungs were found coincident with polyclonal representation with some clonal sub-dominance in expanded Vγ2Vδ2 T cells from lungs of all the unvaccinated macaques. Interestingly, clonal dominance of expanded Vγ2Vδ2 T cells in the lately-infected remote organs such as kidney and liver was associated with undetectable or low-level TB burdens. In fact, while four macaques (2717, 2935, 2823, 3055) exhibited clonal dominance of Vγ2Vδ2 T cells in kidney or liver tissues, we detected no or only a few bacilli organisms in kidney or liver tissues from three of these macaques 2717, 2935, and 2823, and <400 bacilli organisms in liver/kidney tissues from the macaque 3055. However, in the macaque 2722 that did not exhibit clonal dominance, >1300 bacilli organisms were detected in the liver/kidney tissues. Overall, the Mtb burdens in lung tissues were significantly higher than those in liver or kidney tissues(p<0.011). Notably, no TB lesions were seen in kidneys from all macaques, and only a few small non- caseating granulomas were found in the liver tissues from two macaques 2722(no apparent clonal dominance) and 3055, but not in the other three macaques who exhibited clonal dominance in liver tissues. Thus, clonal dominance of expanded Vγ2Vδ2 T cells in the lately-infected organ kidney or liver was associated with undetectable or low-level TB burden.

### Vγ2Vδ2 T cells that accumulated in tissue compartments could mount effector function and produce anti-mycobacterium cytokine

**Figure 5:**
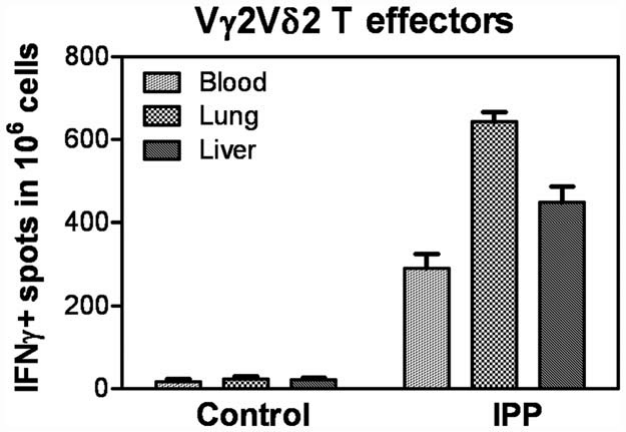
Vγ2Vδ2 T cells that accumulated in tissue compartments could mount effector function and produce anti-TB cytokine. Shown are ELISPOT data for IPP-driven IFNγ+ cellular response in lymphocytes from blood, lung and liver collected from four Mtb-infected macaques at 4–6 weeks after the infection. Data were subtracted from values of glucose/medium control and expressed here as IFNγ+ Vγ2Vδ2 T cells in 10∧6 lymphocytes. IPP stimulates activation of only Vγ2Vδ2 T cells but not other immune cells.

Finally, we asked an interesting question as to whether Vγ2Vδ2 T cells that trafficked to and localized in non-lymphoid tissues such as lung and liver could mount effector function producing cytokines in response to phospholigand. We evaluated antigen-driven production of IFN-γ as this cytokine has been shown to be critical for anti-TB immunity in murine TB model and as Vγ2Vδ2 T effector cells could produce copious amounts of IFN-γ [15]. Interestingly, Vγ2Vδ2 T cells isolated from lung and liver tissues were able to produce IFN-γ in response to phospholigand IPP stimulation *in vitro *(Fig. 5). The numbers of Vγ2Vδ2 T effector cells producing IFN-γ in the tissue compartments were slightly higher than those in PBL (Fig. 5). Thus, these results suggest that significant numbers of Vγ2Vδ2 T cells that localized in the lung and liver had effector function producing anti-TB cytokine, IFN-γ, in response to IPP stimulation.

## Discussion

The TCR repertoire of mycobacterium-specific Vγ2Vδ2 T cells appears to be extremely broad at the level of a single Vδ2 recombination with Jδ/Cδ genes. Most TCR clones found in the lymphoid system, lung, kidney and liver were distinct despite that a few clonotypes in the blood could be repeatedly identified in the spleen, kidney or lung tissues. Notably, all three Jδ segments were employed by Vγ2Vδ2 T cells. In fact, we previously found that even in the down-regulation of γδ T cells during advanced SIVmac infection, TCR repertoires of macaque Vγ2Vδ2 T cells were still quite broad with extremely large pools of distinct TCR clonotypes in the blood [23]. Broad TCR repertoires may be attributed to the repeated DN-DN regions and the unlimited selection of 3 Jδ segments during the TCR development. Functionally, broad TCR repertoires of Vγ2Vδ2 T cells appear to allow these γδ T cells to massively proliferate and expand without constraints. In fact, HMBPP plus IL-2 treatment or mycobacterial infections can rapidly induce up to 400-fold expansion of Vγ2Vδ2 T cells or up to 80% from a baseline level 1% of CD3+ T cells [15], [24].

We have already demonstrated that at 1–2 months after Mtb infection by aerosol route, macaques exhibit increases in numbers of Vγ2Vδ2 T cells in the pulmonary compartment after their proliferation and expansion in lymphoid tissues [11], [18]. In lymphoid tissues and lungs, Vγ2Vδ2 T cells expanded up to 30% from baseline <2% in total CD3+ T cells of the tissues after Mtb infection [18], and numerous Vγ2Vδ2 T cells were infiltrated to lungs and distributed in TB granulomas [18]. Now, molecular analyses of the expanded Vγ2Vδ2 T cells in spleen and lung tissues clearly revealed polyclonal representations of γδ TCR clones and some “clonal sub-dominance” of some Vδ2 clonotypes. Therefore, we interpreted our findings as polyclonal expansion of Vγ2Vδ2 T cells since at least 10–16 unique and representative TCR clones or sequences were identified in expanded Vγ2Vδ2 T cells from lungs and spleens of four of five macaques. We temporally used the term “clonal sub-dominance” in that a number of these TCR clonotypic sequences could sub-dominantly emerge in frequencies 15–20% of all the Vδ2-bearing TCR clones identified in cDNA derived from spleen or lung tissues of the Mtb-infected macaques. The sub-dominance of some Vγ2Vδ2 T-cell clones may be explained by the notion that these clones may express particular phenotypes such as cytokine receptors or memory markers and somehow more favorably proliferate and expand in spleens or lymphoid tissues during immune responses to Mtb infection [25]. On the other hand, other clones may more readily traffic to and accumulate in lung TB granulomas due to expressions of chemokine receptors [15].

One of the novel findings in the current study was that some selected TCR clones could be dominantly present in the expanded Vγ2Vδ2 T cells in kidney without TB lesions or liver with no or subtle TB during a late and subtle Mtb infection of these remote organs. The finding at sequence levels was consistent with the dominance of a selected CDR3 length of Vδ2 junctional regions as revealed by TCR CDR3 spetratyping analyses. Virtually, four of five macaques exhibited such clonal dominance among expanded Vγ2Vδ2 T cells in the remote organ (kidney or liver) after pulmonary Mtb infection by aerosol route, and, interestingly, these macaques exhibited undetectable or low-level Mtb burdens with no or very subtle TB lesions in kidney or liver tissues. This was in contrast to the early-infected lung, in which severe TB lesions and high-level Mtb burdens were coincident with polyclonal representation, rather than clonal dominance, of Vγ2Vδ2 T cells. This finding suggests that TB burdens appear to impact TCR repertoires and tissue trafficking patterns of expanded Vγ2Vδ2 T cells. Polyclonal representation in heavily-infected lungs may result predominantly from simple infiltration or influx due to TB lesions in lung tissues. The mechanism by which clonal dominance of some selected Vγ2Vδ2 T-cell clones in subtly-infected liver or kidney tissues is currently not known. It is likely that low-level Mtb infection without apparent TB lesions in these tissues may provide cytokine or chemokine environment in which to favor trans-endothelial migration of the selected Vγ2Vδ2 T cell clone(s) that express relevant receptors for cytokines/chemokines [26]. This scenario is indeed supported by published human studies demonstrating that some restricted γδ TCR can be detected in gut mucosa [27] and inflammatory kidney tissues of patients with IgA nephropathy [28].

Another interesting observation in the current study was that Vγ2Vδ2 T cells that trafficked to and accumulated in tissue compartments of lung and liver were capable of mounting effector function producing anti-TB cytokine IFNγ in response to phospholigand stimulation *in vitro*. This is in contrast to the speculation that most T cells infiltrating in tissues of inflammatory non-lymphoid organs would be end-terminal or exhausted cells. Our finding suggests that Vγ2Vδ2 T cells accumulated in lung and liver tissues are able to re-recognize phosphoantigen and efficiently mount effector function of IFNγ cytokine production. IFNγ has been shown to play a role in protection against active Mtb infection in mice [5]. While human studies have not found a correlation between blood IFNγ and protection against TB, our recent mechanistic studies suggest that rapid pulmonary trafficking of mycobacterium-specific CD4+ and CD8+ T effector cells producing IFNγ appears to be one of the mechanisms underlying BCG vaccine-induced immunity against primary TB [9].

Our current findings raise an interesting question as to whether timely response of Vγ2Vδ2 T effector cells might indeed contribute to the resistance to TB lesions during late and subtle Mtb infection of liver or kidney tissues after dissemination of pulmonary Mtb infection. We have shown that unlike vaccine-protected macaques, unvaccinated animals infected respiratorily with ∼500 CFU Mtb exhibit significant delays for development and pulmonary trafficking of Ag-specific αβ and γδ T effector cells producing IFNγ and develop severe TB lesions in lungs [8], [9], [11]. Severe TB was associated with transient extrathoracic Mtb dissemination at ∼10–20 days after pulmonary Mtb infection [18], and subsequently led to a late and subtle infection in remote organs (kidney and liver). Activation of Vγ2Vδ2 T cells may be initiated sometime after pulmonary Mtb infection, and be augmented appreciably at the time (days10–20) when a late/subtle infection is anticipated in the kidney/liver. The clonal dominance of expanded Vγ2Vδ2 T cells in kidney or liver suggests that these γδ T cells might efficiently traffic to kidney/liver tissues before Mtb mediates damages or lesions in these organs. It is noteworthy that Vγ2Vδ2 T effector cells could confer homeostatic protection against lung plague lesions [16], and that IFNγ-producing Vγ2Vδ2 T cells were present in “lesions-free” kidney or liver after a late/subtle infection in these remote organs (Figs. 3,5, and [18]). Although current study did not have a power to conclude, our findings provide a rationale to conduct future studies to determine if timely response of Vγ2Vδ2 T effectors plays a role in limiting TB lesions during a late/subtle Mtb infection of the remote organ kidney or liver after initial pulmonary exposure to Mtb.

## Methods

### Ethics statement

The nonhuman primates were used because only primates, but not other species, have TB-specific gamma delta T cells(Vγ2Vδ2 T cells), and because the study cannot be done in humans. The use of macaques and experimental procedures were approved by our Institutional Animal Care and Use Committee (Animal Care Committee), and Institutiaonal Biosafety Committee, and we followed the national and international guidelines regarding “The use of non-human primates in research” to minimize potential suffering of the studied macaques. All the animals were observed 3 times a week and daily after Mtb infection to ensure that animals would not suffer from severe coughing, respiratory distress, weight loss and other potential life-threatening symptoms. Humane euthanization procedures were immediately taken if these symptoms occur or progress. Animals were sedated by Ketamine before sampling, and euthanized by Phentobarbital at the endpoint.

### Macaque animals and *M. tuberculosis* infection

Indian rhesus macaques, 2 years old, were included in these studies. Healthy unvaccinated macaques (animal IDs: Mm2717; Mm2722, Mm3055, Mm2935) were infected with 400–500 CFU Mtb (Rv37 strain) by aerosol route. These macaques were euthanized for gross pathology, bacteriology, and immunology studies at 1–1.5 months after the infection [9], [18]. A BCG-vaccinated macaque (Mm2823) were infected for 2.5-months with Mtb, then received 3-month daily treatment with anti-TB drugs[ isoniazid (5 mg/kg) and pyrazinamide (15 mg/kg) mixing with yogurt as previously described [8] ], and finally re-infected with Mtb again by aerosol. Complete necropsy studies were done one month after Mtb re-infection. All the animal protocols for the studies were IACUC-approved.

### Isolation of single cell suspensions and lymphocytes from blood, lymphoid tissues, and non-lymphoid tissues from the rhesus macaques

PBL were isolated from EDTA blood of the monkeys using Ficoll/diatrizoate gradient centrifugation. Spleen tissues were carefully teased to generate single-cell suspensions. Tissue pieces from lungs, livers, and kidneys were minced in RPMI medium, as previously described [19], [29], to collect single cell suspensions (mainly lymphocytes and tissue macrophages). The single cells suspensions from these non-lymphoid organs were divided into three parts: one directly used for mycobacterial CFU counts [8]; one directly saved as pellets for real time quantitation of *M. tuberculosis* Ag85B RNA [18]; one subjected to isolation of lymphocytes by Ficoll/diatrizoate gradient centrifugation for flow cytometry-based analyses of γδ T cells and molecular studies of γδ TCR repertoires.

### Flow cytometry analyses of Vγ2Vδ2 T cells

Rhesus lymphocytes isolated from the blood and lymphoid and non-lymphoid tissues were stained immunologically with anti-Vγ2, anti-Vδ2, anti-Cδ (Pan γδ), and anti-CD3 antibodies, as described previously [22]. Isotype-matched Ig or anti-Vδ3 Ab in combination with other antibodies served as controls as previously described [22].

### ELISPOT measuring of phosphoantigen-specific IFNγ-producing Vγ2Vδ2 T cells

The assay was done as previously described [9], [18]. Phosphoantigen isopentenyl pyrophosphate (IPP) was purchased from Sigma (St Louis), and used at the working concentration of 15 um/L. The justification is that IPP is recognized only by Vγ2Vδ2 T cells but not other immune cells.

### Bacterial colony forming units (CFU) counts

Mtb infection levels in the blood, lung cells and other tissue cells were determined by the quantitation of bacillus CFUs in cell lysates from tissues cells of Mtb-infected macaques, as previously described [8], [11], [18].

### The real time quantitative PCR for quantitation of *M. tuberculosis* Ag85B mRNA

This was done as previously described [8], [18].

### Gross and microscopic analyses of TB lesions

Semi-quantitative pathology analyses were done as previously described [8], [9], [18].

#### Isolation of RNA from cells and cDNA synthesis

Total RNA was isolated from PBL or lymphocytes isolated from spleen, lung, liver and kidney tissues of the infected macaques using the TRIzol isolation method [30]. The RNA pellet was resuspended in RNase-free deionized water and then used immediately for synthesis of cDNA using the protocol provided in the cDNA synthesis kit from Clontech Laboratories (Palo Alto, CA).

### Cloning and sequencing of Vδ2-bearing TCR

Molecular cloning and sequencing of Vδ2^+^ T cells were done using a PCR-based technique as described previously [11], [23]. cDNA derived from PBL obtained before and after Mtb infection as well as from lymphocytes isolated from lung, liver and kidney tissues of the infected macaques. We used these tissue lymphocytes for studies of γδ TCR repertoires because marked expansions of Vγ2Vδ2 T cells were identified in the infected macaques. Vδ2 TCR cDNA was then amplified by a 30-cycle PCR using a pair of Vδ2- and Cδ-specific primers that bear EcoRI and XbaI restriction sites, respectively. The sequences for the primers were as follows: Vδ2,5′-GCGCGAATTCAACGGATGGTTTGGTATGAGG-3′; Cδ,5′-GCGCTCTAGATATATCAACTGGTACAGG-3′. The specific PCR products were gel-purified, digested with EcoRI and XbaI and ligated into the plasmid SP65 (Promega, Madison, Wisconsin) for cloning and sequencing.

#### Strategy for sequencing and frequency analyses of TCR cDNA clones

Given that Vδ2 TCR repertoire were quite diverse even in SIVmac-infected macaques [23], we initially sequenced 25 TCR clones isolated from cDNA derived from Vγ2Vδ2 T cells. If the frequency for one or two of clonotypes was >20% of all clones analyzed, additional 20–25 clones were sequenced and analyzed.

### TCR CDR3 spetratyping of Vδ2^+^ T cells

CDR3 profiles were analyzed by Genescan-based spectratyping as we previously described [22]. cDNAs were amplified by PCR for expression of Vδ2 family gene using a Vδ2-specific primer (5′-GGGGACCCTGCCACCCTCAAGTGC-3′) and a Cδ-specific primer (5′-CTTGGGGTAGAAGTCCTTCAC-3′). A second round of PCR was performed using an internal Vδ2 primer (5′-ATGAAAGGAGAAGCAATCAGTAAC-3′) and an internal Cδ primer (5′-CAGACAAGCAACATTTGTCCC-3′). The internal Cδ primer was labeled at its 5′ end with the Fam fluorophore (Applied Biosystems, Foster City, CA), designed as previously described [22]. The first and second round PCR were amplified for 35 and 15 cycles, respectively, using the following conditions: 95°C for 30 seconds, 60°C for 30 seconds, and 72°C for 30 seconds. One micro liter of each reaction product was mixed with deionized formamide and a ROCK-500 size standard, and then electrophoresed on a 5% acrylamide gel on a 310 DNA sequencer (Applied Biosystems, Foster City, CA). Data were analyzed for size and fluorescence intensity using the Genescan software. These lengths were expressed as predicted numbers of amino acids [22].

### Statistical analysis

The multivariate analysis of variance (ANOVA) and student *t* test were used, as previously described [29], to statistically analyze the data for differences in Vγ2Vδ2 T cells numbers or *M. tuberculosis* burdens between tissues/organs. We also employed two-tailed Fisher exact test, as previously described [20], [21], to examine whether there were significant differences in dominant Vδ2 clonotypes (perturbation of TCR repertoire) between blood, spleen, lung, liver and kidney tissue compartments. Any clones comprising ≥20% of the clones identified in a tissue compartment of a macaque were defined as dominant Vδ2 clonatypes, and the frequencies of these dominant Vδ2 clonotypes in a given tissue compartment from total five macaques were calculated and compared statistically with those in individual different tissue compartments. We also statistically compared percentage numbers of total distinct Vδ2-bearing clones between different tissues compartments (n = 5),
